# Differential CpG DNA methylation of peripheral B cells, CD4^+^ T cells, and salivary gland tissues in IgG4-related disease

**DOI:** 10.1186/s13075-022-02978-5

**Published:** 2023-01-07

**Authors:** Xunyao Wu, Anqi Wang, Mu Wang, Yu Peng, Yingying Chen, Jieqiong Li, Zheng Liu, Hui Lu, Jiaxin Zhou, Linyi Peng, Yan Zhao, Xiaofeng Zeng, Yunyun Fei, Wen Zhang

**Affiliations:** 1 Department of Rheumatology and Clinical Immunology, Peking Union Medical College Hospital, Chinese Academy of Medical Sciences & Peking Union Medical College, Beijing, China; 2 Clinical Biobank, Department of Medical Research Center, Peking Union Medical College Hospital, Chinese Academy of Medical Sciences & Peking Union Medical College, Beijing, China; 3 National Clinical Research Center for Dermatologic and Immunologic Diseases (NCRC-DID), Ministry of Science & Technology; State Key Laboratory of Complex Severe and Rare Diseases, Peking Union Medical College Hospital, Chinese Academy of Medical Sciences & Peking Union Medical College, Beijing, China; 4 Key Laboratory of Rheumatology & Clinical Immunology, Ministry of Education, Beijing, 100730 China; 5 Department of Stomatology, Peking Union Medical College Hospital, Chinese Academy of Medical Sciences & Peking Union Medical College, Beijing, China

**Keywords:** IgG4-RD, DNA methylation, B cells, CD4^+^ T cells, Salivary gland tissues

## Abstract

**Objectives:**

Immunoglobulin-G4-related disease (IgG4-RD) is a distinct systemic autoimmune-mediated disease manifesting as chronic inflammation and tissue fibrosis. Since the role of DNA methylation in the pathogenesis of IgG4-RD is still unclear, we conduct this study to investigate epigenetic modifications in IgG4-RD.

**Methods:**

A genome-wide DNA methylation study was conducted with B cells, CD4^+^ T cells, and salivary gland tissues from IgG4-RD patients and matched controls by using the Illumina HumanMethylation 850K BeadChip. We further performed pyrosequencing and immunohistochemistry assays to validate the methylation status of some targets of interest.

**Results:**

We identified differentially methylated CpG sites including 44 hypomethylated and 166 hypermethylated differentially methylated probes (DMPs) in B cells and 260 hypomethylated and 112 hypermethylated DMPs in CD4^+^ T cells from 10 IgG4-RD patients compared with 10 healthy controls. We also identified 36945 hypomethylated and 78380 hypermethylated DMPs in salivary gland tissues of 4 IgG4-RD patients compared with 4 controls. DPM2 (cg21181453), IQCK (cg10266221), and ABCC13 (cg05699681, cg04985582) were hypermethylated and MBP (cg18455083) was hypomethylated in B cells, CD4^+^ T cells, and salivary gland tissues of IgG4-RD patients. We also observed the hypomethylated *HLA-DQB2* in CD4^+^ T cells from IgG4-RD patients. Kyoto Encyclopedia of Genes and Genomes (KEGG) pathway analysis of DMPs in salivary gland tissues of IgG4-RD patients revealed enrichment of pathways involved in the regulation of immune cell responses and fibrosis.

**Conclusion:**

This is the first DNA methylation study in peripheral B cells, CD4^+^ T cells, and salivary gland tissues from IgG4-RD patients. Our findings highlighted the role of epigenetic modification of DNA methylation and identified several genes and pathways possibly involved in IgG4-RD pathogenesis.

**Supplementary Information:**

The online version contains supplementary material available at 10.1186/s13075-022-02978-5.

## Introduction

Immunoglobulin-G4-related disease (IgG4-RD) is a distinct systemic autoimmune-mediated disease characterized by the infiltration of IgG4+ plasma cells into one or multiple organs and the development of tumor-like masses [1]. Chronically activated B cells, T cells, and macrophages accumulate in fibrotic lesions of IgG4-RD [2]. Moreover, IgG4-RD has an estimated incidence of 0.28–1.08 per person, with 336-1,300 newly diagnosed patients per year [3]. Systemic corticosteroid administration and B cell depletion are the main therapeutic strategies, and both could significantly ameliorate IgG4-RD. However, because of glucocorticoid-induced toxicity and high relapse rate after rituximab treatment [4], IgG4-RD pathogenesis needs to be further explored.

Epigenetic mechanisms are found to be related to the risk of various autoimmune diseases [5–7]. DNA methylation is a well-studied epigenetic modification involved in gene expression regulation [8]. So far, numerous studies have provided evidence of aberrant DNA methylation profiles in rheumatoid arthritis (RA), systemic lupus erythematosus (SLE), and primary Sjögren's syndrome (pSS) [9–18]. However, changes in DNA methylation of IgG4-RD patients are poorly understood.

In this study, B cells and CD4^+^ T cells were purified from 10 IgG4-RD patients and 10 age- and sex-matched healthy controls (HCs). DNA methylation was examined using a genome-wide Illumina 850K CpG promoter. We also performed DNA methylation analysis of salivary gland tissues biopsied from 4 IgG4-RD patients and 4 HCs. Our findings demonstrated the pattern of DNA methylation in peripheral B cells, CD4^+^ T cells, and salivary gland tissues involving key genes and pathways associated with IgG4-RD pathogenesis.

## Materials and methods

### Study cohort and ethics

A total of 20 IgG4-RD patients were enrolled at Peking Union Medical College Hospital between 2020 and 2022 in our study. The diagnosis of IgG4-RD was established according to the 2019 American College of Rheumatology/European League Against Rheumatism classification criteria and the 2011 comprehensive diagnostic criteria for IgG4-RD [19, 20]. Peripheral blood samples were obtained from IgG4-RD patients and age-, sex-, and ethnicity-matched healthy blood donors. Salivary gland tissues were obtained from individuals examined for a possible IgG4-RD diagnosis. The biopsy specimens that showed no inflammation and negative serology were considered as case controls. Demographic and clinical characteristics of the study participants are presented in Supplementary Table 1. The diagnostic score was calculated according to the 2019 American College of Rheumatology/European League Against Rheumatism classification criteria for IgG4-RD. Disease activity was assessed on the basis of the IgG4-RD responder index [21].

This study was approved by the Ethics Board of Peking Union Medical College Hospital (No. JS-3389). All participants signed a written informed consent.

### Isolation of B cells and CD4^+^ T cells

Peripheral blood mononuclear cells (PBMCs) were isolated from fresh peripheral blood samples through density gradient centrifugation (Ficoll-Paque, Cat. No. 7111011, DAKEWE, China). CD4^+^ T cells and B cells were separated from PBMCs using CD4 MicroBeads, human (Cat. No. 130-045-101, Miltenyi Biotec, Cambridge, MA) and B Cell Isolation Kit II (Cat. No. 130-091-151, Miltenyi Biotec, Cambridge, MA), respectively, according to the manufacturer’s protocol. The purity of isolated CD4^+^ T cells and B cells was confirmed to be >95% by flow cytometry.

### DNA methylation profiling

DNA was extracted from cells or tissues using DNeasy Blood & Tissue Kit (Cat. No. 69506, Qiagen, Germany). The purity and concentration of DNA was measured using Nanodrop 2000 (ThermoScietific). Approximately, 500 ng of genomic DNA from each sample was used for sodium bisulfite conversion using the EZ DNA Methylation-Gold Kit (Cat. No. D5006, Zymo Research, USA). Genome-wide DNA methylation was assessed with the Illumina Infinium Human Methylation 850K BeadChip (Illumina Inc, USA) according to the manufacturer’s instructions. These steps were performed by Novogene Company (Beijing, China).

### Analysis of DNA methylation data

DNA methylation files were processed in the R environment using the Minfi statistical analysis package (V1.30.0) for background signal correction and normalization of each channel to control probes included in each array. For each sample, (1) CpG sites with a *p* value of >0.01 and (2) probes with single nucleotide polymorphisms or their single base extension and X and Y chromosomes at the CpG site were excluded from the analysis [22–24].

The methylated and unmethylated probe values were used to calculate the β value ratio according to the following formula: *β* value = (methylated)/(methylated + unmethylated + 100). The mean difference *β* value (Δβ) and the adjusted *p* value between HCs and IgG4-RD patients for each CpG site were calculated using CHAMP packages (V2.14.0) [22]. For B cells or CD4^+^ T cells isolated from PBMCs, the adjusted p value ≤ 0.05 or Δβ > 0.05 was considered statistically significant. For tissue samples, the adjusted *p* value < 0.05 and Δβ ≥ 0.2 was considered statistically significant [25–29]. Differential methylation regions (DMRs) were analyzed using the DMRcate package (V1.20.0), with FDR-corrected p values < 0.05 being considered statistically significant.

### Pathway analysis

Statistically significant gene lists were used to perform the Kyoto Encyclopedia of Genes and Genomes (KEGG) and Gene Ontology (GO) analyses. The ClusterProfiler package (V3.12.0) was used for pathway enrichment analysis [30].

### Pyrosequencing

DNA of purified cells and tissues was extracted using a QIAamp DNA Mini Kit (Cat.No. 51304, Qiagen, Germany) and then bisulfite-modified with the Qiagen EpiTect Bisulfite Kit (Cat.No. 59104, Qiagen, Germany). Primers were designed using PyroMark Assay Design 2.0 (Qiagen, Germany) and presented in Supplementary Table 9. PCR amplification of the target region was performed using the KAPA2G Robust HotStart PCR kit (Cat. No. KK5525, Roche) on the ABI 9700 PCR System (Applied Biosystems, USA) to produce biotinylated PCR amplicons required for the pyrosequencing reaction. After bisulfite treatment and PCR, the substrate mixture, enzyme mixture, and four deoxynucleoside triphosphate (dNTP) (Cat.No. 201900, Qiagen, Germany) were successively added for the reaction on the pyrosequencing detector (PyroMark Q96 ID, Qiagen, Germany). The methylation status of each site was automatically analyzed using the Pyro Q—CpG software (Qiagen, Germany).

### Immunohistochemistry

Deparaffinated sections of salivary glands from IgG4-RD patients and controls were immersed in Tris-EDTA antigen retrieval solution, pH 9.0 (Cat.No. G1203, Servicebio, Wuhan, China) and microwaved for antigen retrieval. Then sections were incubated in 3% hydrogen peroxide for 25 min to quench endogenous peroxidase activity, followed by tissues uniformly covered with 3% BSA (Cat.No. GC305010, Servicebio) at room temperature for 30 min. Diluted primary antibody anti-Myelin Basic Protein antibody (dilution 1:400, ab7349, Abcam) was added to sections and incubated overnight at 4°C. After incubating with the HRP-labeled secondary antibody (dilution 1:200, Cat.No. GB23302, Servicebio) at room temperature for 50 min, sections were reacted with DAB (3,3-N-Diaminobenzidine) solution (Cat.No. G1212, Servicebio) for 5 min, and counterstained with hematoxylin (Cat.No. G1004, Servicebio) for 3 min.

## Results

### Global DNA methylation patterns in peripheral blood and salivary gland tissues from IgG4-RD patients and controls

**Fig. 1:**
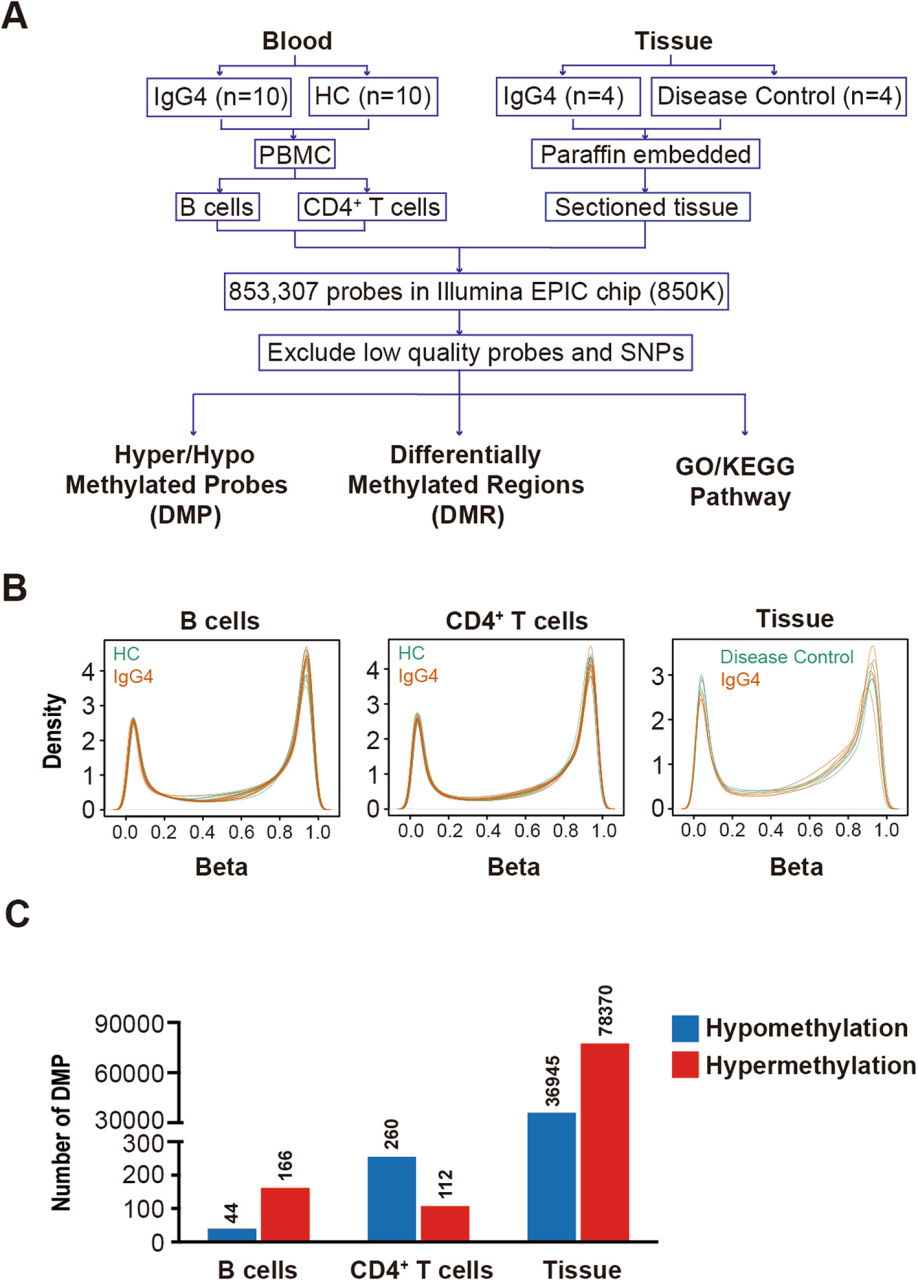
Genome-wide DNA methylation patterns in IgG4-RD patients. **A** Graphical overview of the study design. **B** Distribution of methylation beta values in B cells, CD4+ T cells, and salivary gland tissues of IgG4-RD patients and controls. **C** Number of hypomethylation and hypermethylation probes in B cells, CD4+ T cells, and salivary gland tissues of IgG4-RD patients compared with controls

The DNA methylation profile of B cells and CD4^+^ T cells from 10 HCs and 10 IgG4-RD patients was analyzed. The DNA methylation profile of salivary gland tissues from 4 IgG4-RD patients and 4 controls was also assayed. The workflow is presented in Fig. 1A. The overall DNA methylation beta value displayed similar distribution patterns between the two groups (Fig. 1B). 44 and 166 sites were hypomethylated and hypermethylated, respectively, in B cells (Fig. 1C). Moreover, 260 and 112 sites were hypomethylated and hypermethylated, respectively, in CD4^+^ T cells from IgG4-RD patients compared with HCs (Fig. 1C). In total, 36,945 sites were hypomethylated and 78,370 sites were hypermethylated in salivary gland tissues of IgG4-RD patients compared with HCs (Fig. 1C).

**Fig. 2:**
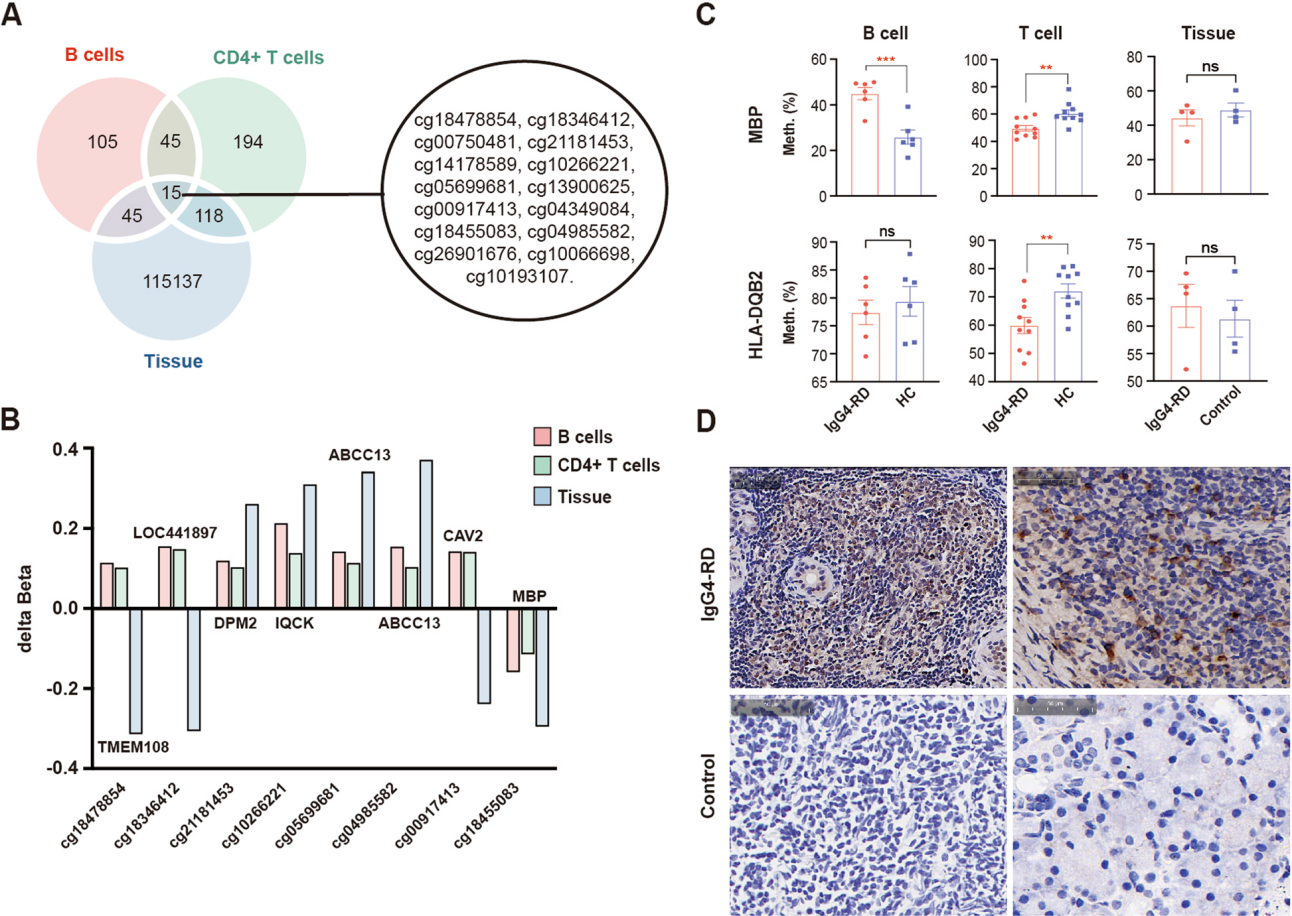
Overlapped DNA methylation profiles in B cells, CD4^+^ T cells, and salivary gland tissues of IgG4-RD patients. **A** Venn diagram showing overlapped differentially methylated CpG sites in B cells, CD4^+^ T cells, and salivary gland tissues of IgG4-RD patients. **B** Delta beta values of overlapped differentially methylated genes and CpG sites in B cells, CD4^+^ T cells, and salivary gland tissues of IgG4-RD patients. **C** The methylation rate of MBP and HLA-DQB2 in B cells, CD4+ T cells, and salivary gland tissues of IgG4-RD patients compared with controls through pyrosequencing. **D** Immunohistochemistry staining of MBP in salivary gland tissues of two IgG4-RD patients and two controls (scale: 50μm)

Next, genes and differentially methylated probes (DMPs) of salivary gland tissues were obtained and compared with those of B cells and CD4^+^ T cells in IgG4-RD patients. As presented in Fig. 2A, 15 DMPs were common to all three cell types. To further identify the methylation status of common genes in the three cell types, the delta beta value was calculated. DPM2 (cg21181453), IQCK (cg10266221), and ABCC13 (cg05699681, cg04985582) were hypermethylated and MBP (cg18455083) was hypomethylated in B cells, CD4^+^ T cells, and salivary gland tissues of IgG4-RD patients compared with HCs (Fig. 2B). Although TMEM108 (cg18478854), LOC441897 (cg18346412) and CAV2 (cg00917413) were hypermethylated in both B cells and CD4^+^ T cells, they were hypomethylated in salivary gland tissues of IgG4-RD patients compared with HCs (Fig. 2B).

We further performed pyrosequencing in B cells, CD4^+^ T cells, and salivary gland tissues from another cohort consisted of 10 IgG4-RD patients and 10 age-, sex-, and ethnicity-matched healthy blood donors, to validate the methylation state of MBP and HLA-DQB2. In line with the results obtained by DNA methylation chip, the methylation rate of MBP and HLA-DQB2 in CD4^+^ T cells was significantly lower in IgG4-RD patients than in HCs (Fig. 2C). Although a tendency of lower DNA methylation rate of MBP was observed in salivary gland tissues of IgG4-RD patients compared with controls, no statistical difference was found (Fig. 2C). Immunohistochemical staining showed a high expression of MBP in salivary gland tissues of IgG4-RD patients, which further supported the hypomethylated status of MBP (Fig. 2D).

### DNA methylation status of B cells from IgG4-RD patients

**Fig. 3:**
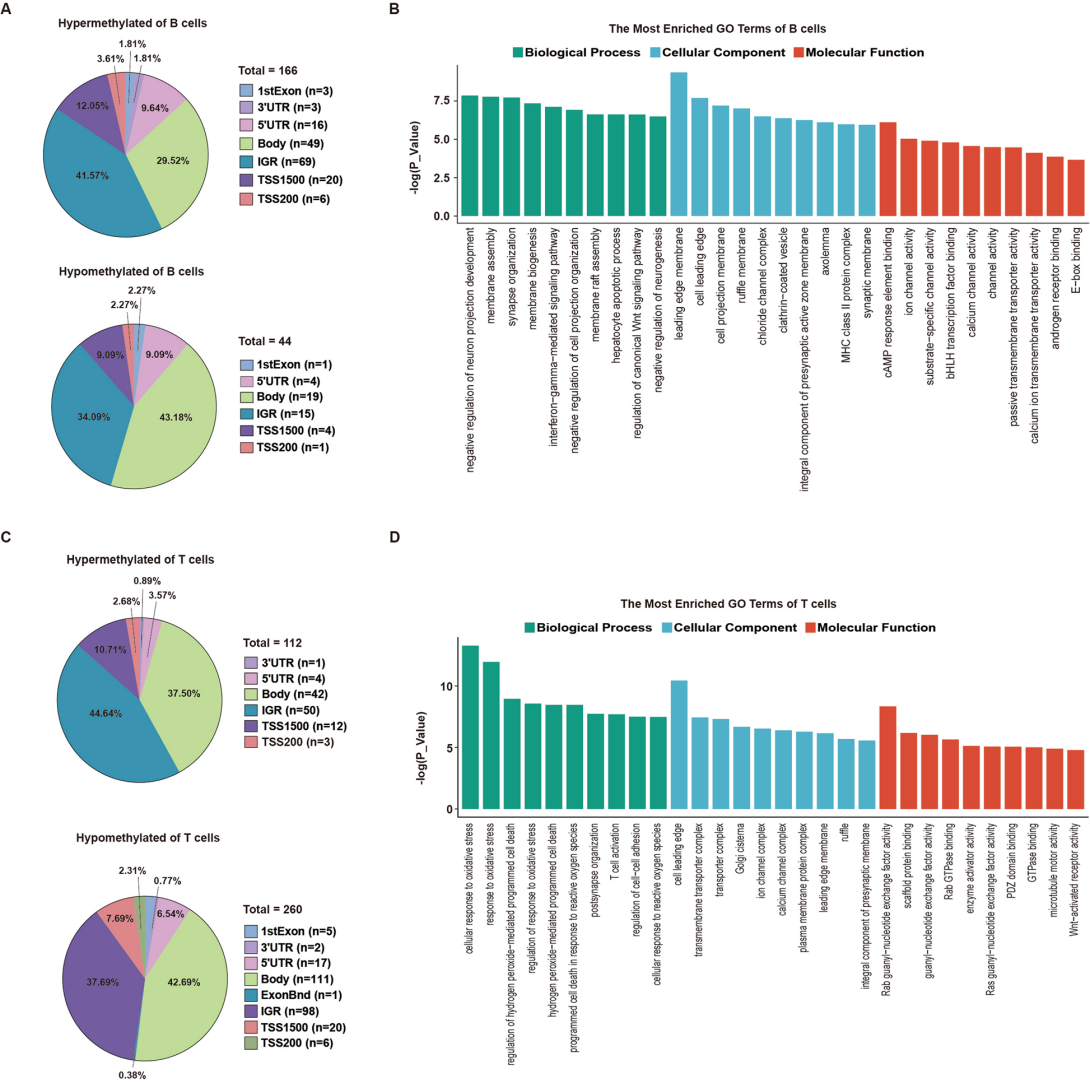
DNA methylation status in B cells and CD4^+^ T cells of IgG4-RD patients. **A** Distribution of DMPs in B cells of IgG4-RD patients. **B** Significant GO categories in B cells of IgG4-RD patients. **C** Distribution of DMPs in CD4^+^ T cells of IgG4-RD patients. **D** Significant GO categories in CD4^+^ T cells of IgG4-RD patients

Next, we analyzed B cells from 10 IgG4-RD patients and 10 HCs and identified 166 hypermethylated and 44 hypomethylated probes (Fig. 3A). Among them, most DMPs were present within the intergenic regions (IGRs) or the gene body (Fig. 3A). Table S2 and Table S3 present the top associated hypermethylated and hypomethylated probes with annotated genes, respectively. *IQCK*, *UMODL1*, *AHDC1*, *LARS2*, *RASA3*, *USP16*, *BTBD11*, *CLECL1*, *LOC441897*, and *ABCC13* were hypermethylated while *TMEM9B*, *CLIC6*, *MIR492*, *ARNTL*, *TNN*, *MBP*, *CDK15*, *PIGL*, *HLA-DRB1*, and *MYO1D* were hypomethylated at CpG sites in B cells from IgG4-RD patients.

GO analysis of the DMPs revealed enrichment for multiple categories. Among them, the interferon-gamma-mediated signaling pathway (related genes: *TRIM68*, *HLA-DRB1*, *HLA-DQB2*, and *IFNGR2*) and regulation of the canonical Wnt signaling pathway (related genes: *STK4*, *BICC1*, *KANK1*, *ARNTL*, *TNN*, and *PSMD5*) were found to be enriched (Fig. 3B).

### DNA methylation status of CD4^+^ T cells from IgG4-RD patients

Furthermore, we examined CD4^+^ T cells from 10 IgG4-RD patients and 10 HCs and identified 112 hypermethylated and 260 hypomethylated probes (Fig. 3C). Similar to B cells, most DMPs of CD4^+^ T cells were present within the IGRs or the gene body (Fig. 3C). Table S4 and Table S5 present the top associated hypermethylated and hypomethylated probes with annotated genes. *MAST4*, *AHDC1*, *BICC1*, *USP16*, *IQCK*, *LOC727677*, *MYO1D*, *LOC441897*, *CAV2*, and *TGFBR2* exhibited hypermethylation while *TMEM9B*, *CLIC6*, *CCS*, *PAWR*, *FIP1L1*, *ARHGAP15*, *ARHGAP26*, *PKD1L1*, *HLA-DRB1*, and *HLA-DQB2* exhibited hypomethylation at CpG sites in CD4^+^ T cells from IgG4-RD patients. Pyrosequencing validated the hypomethylated status of MBP and *HLA-DQB2* in CD4^+^ T cells of IgG4-RD patients compared with HCs (Fig. 2C).

GO analysis of the DMPs revealed enrichment for multiple categories. Among them, cellular response to oxidative stress (related genes: *FUT8*, *ARNTL*, *CFLAR*, *PTPRK*, *ETS1*, *TRAP1*, *FOXO1*, *OXR1*, *PAWR*, *PRR5L*, *CCS*, *PYROXD1*, and *MIR21*), T cell activation (related genes: *CLECL1*, *TGFBR2*, *ZMIZ1*, *EOMES*, *CD3E*, *TIGIT*, *CLEC7A*, *PAWR*, *KIF13B*, *CD300A*, *FZD7*, and *MIR21*), and Wnt-activated receptor activity (related genes: *LRP5* and *FZD7*) were found to be enriched (Fig. 3D).

### Identified DMPs and DMRs in salivary gland tissues of IgG4-RD patients

**Fig. 4:**
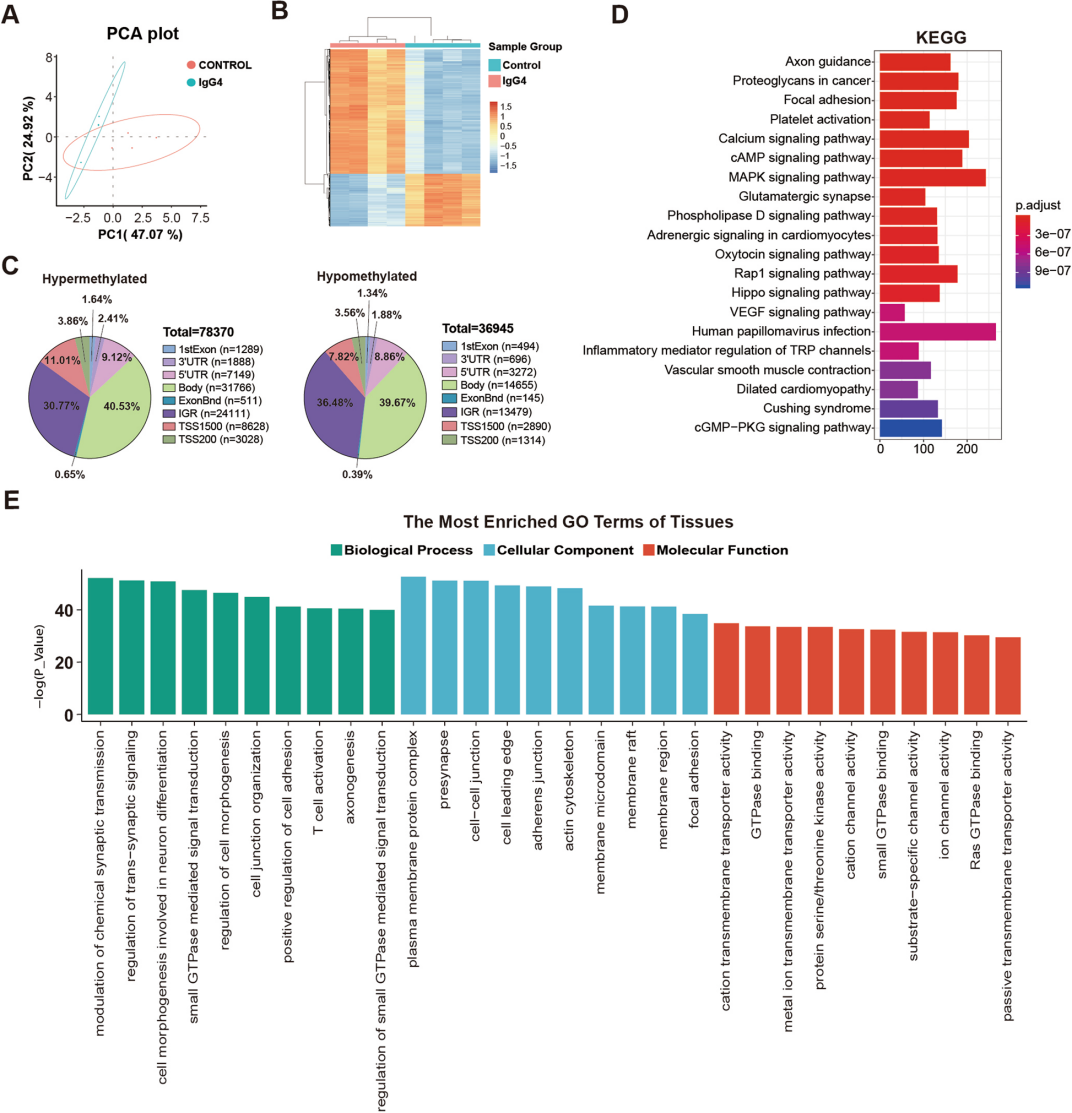
DNA methylation status in salivary gland tissues of IgG4-RD patients. **A** Principal components analysis of DMPs. Ellipses show the 95% confidence interval for the distribution of each group. Each dot represents an individual patient. **B** Supervised hierarchical clustering of DNA methylation in salivary gland tissues of IgG4-RD patients. **C** Distribution of DMPs in salivary gland tissues of IgG4-RD patients. **D** KEGG pathway analysis of DMPs in salivary gland tissues of IgG4-RD patients. **E** Significant GO categories in salivary gland tissues of IgG4-RD patients

Furthermore, we investigated the DNA methylation status in the affected organ by using the salivary gland tissues of IgG4-RD patients. In total, 78370 hypermethylated and 36945 hypomethylated probes were identified in the salivary gland tissues of IgG4-RD patients compared with controls (Fig. 4A–C). Among them, most DMPs were also present within the IGRs or the gene body (Fig. 4C). Table S6 and Table S7 present the top 25 associated hypermethylated and hypomethylated probes with annotated genes. *LOH12CR1*, *PFKP*, *ARSB*, *SNX29*, *TBCD*, *STARD13*, *GALNT10*, *EIF4E3*, *VGLL4*, *TGFBR3*, *GLG1*, *RBM34*, *EPAS1*, *ITPKB*, *TBS22D1*, *SLC44A2*, *TRIOBP*, *ACAD11*, *KCNAB2*, *PDE4D*, *RERE*, *ARID1B*, *UAP1*, *LGALS3*, and *LOC102724933* genes exhibited hypermethylation. *CD69*, *RUNX1*, *TMEM229B*, *JAZF1*, *SLC7A6*, *FAM69A*, *EVL*, *CYTIP*, *WIPF1*, *TNFSF8*, *LOC102724*, *NCKAP1L*, *SETBP1*, *KIAA0748*, *PPP1R16B*, *GRAP2*, *CASP8*, *ARSG*, *ITGB2-AS1*, *MGAT1*, *CD37*, *MAP2K2*, *TNFAIP8L2*, and *RCSD1* exhibited hypomethylation at CpG sites in the salivary gland tissues of IgG4-RD patients.

The KEGG pathway analysis identified the pathways whose genes from the salivary gland tissues of IgG4-RD patients were differentially methylated: “calcium signaling pathway,” “cAMP signaling pathway,” “MAPK signaling pathway,” “oxytocin signaling pathway,” “Rap1 signaling pathway,” “Hippo signaling pathway,” “VEGF signaling pathway,” and “cGMP-PKG signaling pathway” (Fig. 4D). The GO analysis of the DMPs revealed enrichment for the positive regulation of cell adhesion and T cell activation (Fig. 4E).

**Fig. 5:**
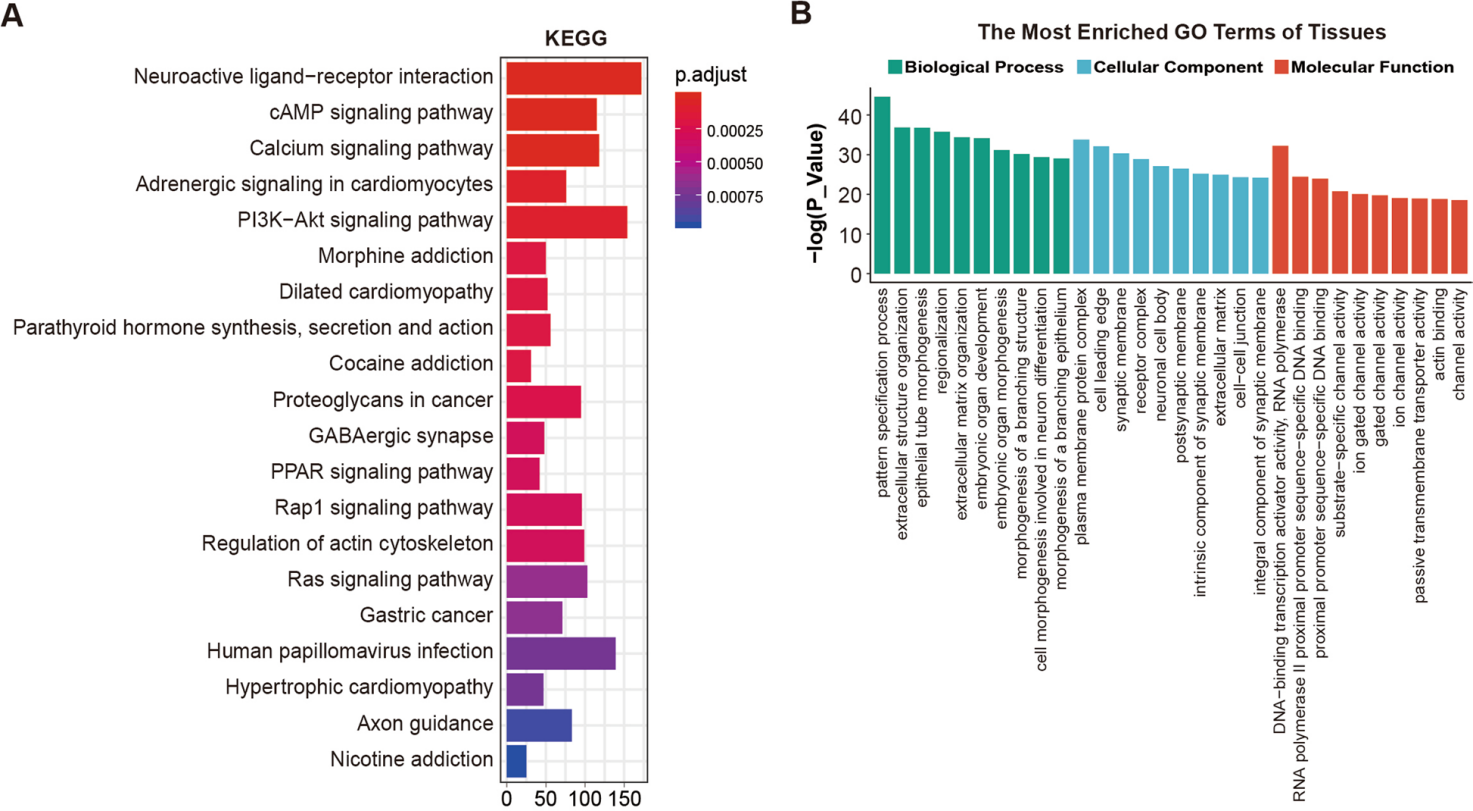
Enrichment of DMR-related genes in salivary gland tissues of IgG4-RD patients. **A** KEGG pathway analysis of DMR-related genes in salivary gland tissues of IgG4-RD patients. **B** Significant GO categories of DMR-related genes in salivary gland tissues of IgG4-RD patients

Finally, we characterized the most significant DMRs in the salivary gland tissues of IgG4-RD patients (Table S8). For the better characterization of genes detected in the DMRs, GO and KEGG pathway analyses were performed. The KEGG analyses identified genes involved in the cAMP signaling pathway, calcium signaling pathway, PI3K-Akt signaling pathway, PPAR signaling pathway, Rap1 signaling pathway, and Ras signaling pathway (Fig. 5A). The GO analyses revealed genes most enriched in extracellular matrix organization and ion channer activity (Fig. 5B).

## Discussion

Currently, epigenetic modifications in IgG4-RD are poorly understood. We here performed a genome-wide DNA methylation analysis of peripheral B cells and CD4^+^ T cells, as well as salivary gland tissues from IgG4-RD patients. A set of DMPs, DMRs, and involved pathways were identified in IgG4-RD patients compared with HCs. Our observations related to DNA methylation patterns provided new insights into epigenetic modifications of peripheral immune cells and tissue-specific differences possibly involved in IgG4-RD pathogenesis.

MBP (cg18455083) was hypomethylated in CD4^+^ T cells and salivary gland tissues of IgG4-RD patients. MBP encodes for myelin basic protein, which is the major target of T cells in lesions of multiple sclerosis (MS) patients and the animal model of experimental autoimmune encephalomyelitis [31]. Numerous studies have demonstrated the role of myelin reactivity of T cells in MS [32–34]. A recent study also found that myelin-specific CD8^+^ T cells exacerbated brain inflammation in central nervous system autoimmunity [35]. Currently, literature about whether myelin has a role in IgG4-RD pathogenesis is lacking. Since CD4^+^ cytotoxic T lymphocytes have been demonstrated to be clonally expanded in the peripheral blood of IgG4-RD patients and implicated in IgG4-RD pathogenesis [36, 37], exploring whether myelin-specific T cells contribute to IgG4-RD development in our future study will be interesting.

A study by a Japanese group suggested the involvement of the HLA class in IgG4-related pancreatitis [38]. Another study by another Japanese group found six HLA class-associated variants, namely rs1143146 (*HLA-A*), rs1050716 (*HLA-C*), c.759_763delCCCCCinsTCCCG (*HLA-C*), rs1050451 (*HLA-C*), rs4154112 (*HLA-DQB1*), and rs1049069 (*HLA-DQB1*), to be associated with the relapse of IgG4-related pancreatitis [39]. In our study, we also found the hypomethylated status of *HLA-DQB2* in CD4^+^ T cells from IgG4-RD patients.

In the present study, several differentially methylated genes with crucial roles in the immune cell regulation or fibrosis were identified in the salivary gland tissues of IgG4-RD patients. *GALNT10* encodes for polypeptide N-acetylgalactosaminyltransferase 10, which is associated with the increased regulatory CD4^+^ T cell infiltration and decreased granzyme B expression in CD8^+^ T cells in tumor tissues [40]. *TGFBR3* encodes for the TGF-beta type III receptor (TGFBR3), an essential constituent of TGF-beta signaling, that regulates both immune responses and fibrosis [41, 42]. *ITPKB* encodes for inositol-trisphosphate 3-kinase B, which is important for B cell survival, development, and function as well as T cell maturation in the thymus [43, 44]. *LGALS3* encodes for galectin-3, which has been implicated in various autoimmune diseases through modulation of T cell functions [45, 46]. *CD69* encodes for CD69, an activated marker for B and T cells that is pivotal in autoimmunity [47]. *RUNX1* encodes for Runt-related transcription factor 1 (RUNX1), a transcription factor that regulates fibrosis [48]. *CD37* encodes for CD37, a B-cell surface antigen widely expressed on mature B cells [49]. The tetraspanin CD37 was recently found to orchestrate the α4β1 integrin-Akt signaling axis and support longer plasma cell survival [50].

The KEGG pathway analysis revealed enrichment of the “calcium signaling pathway,” “MAPK signaling pathway,” and “Hippo signaling pathway” in the salivary gland tissues of IgG4-RD patients. Chronic calcium signaling in IgE^+^ B cells restricts plasma cell differentiation and survival [51]. A recent study found that intraglandular injection of IL-4 into mouse submandibular glands could promote fibrogenesis in IgG4-related sialadenitis through ROS-p38 MAPK-p16^INK4A^ [52]. Although literature about the role of the Hippo signaling pathway in IgG4-RD development is lacking, the yes-associated protein and transcriptional coactivator with PDZ-binding motif, which are transcriptional effectors of the Hippo pathway, have been identified as key matrix stiffness-regulated coordinators of fibroblast activation and matrix synthesis [53].

A previous study identified 55 genes in CD4^+^ T cells and 1628 genes in B cells with differentially methylated CpGs from the peripheral blood of SLE patients. They displayed marked hypomethylation in interferon-regulated genes. Especially, differentially methylated CpGs in B cells were predominantly hypermethylated, and the essential upstream regulators included *TNF* and EP*300* [54]. An integrative analysis of CD4^+^ and CD8^+^ T cells from the multiple-autoimmune disease methylation, including Graves’ disease (GD), RA, SLE, and systemic sclerosis (SSc), demonstrated that hypomethylation of IFN-related genes is a common feature of autoimmune diseases in CD4^+^ T cells [55]. Only 119 probes across 74 genes in purified peripheral CD4^+^ T cells were detected to be differentially methylated in pSS patients compared with controls, while in B lymphocytes, a total of 6707 differentially methylated probes across 3619 genes were identified. Moreover, methylation alteration in B cells was more frequent in interferon-regulated genes in patients who were autoantibody positive [56]. Hypomethylation of interferon (IFN)-regulated genes in B cells and minor salivary gland biopsies of pSS patients were also identified in another study [18]. In our study, both B cells and CD4+ T cells did not display marked differentially methylated CpGs from the peripheral blood of IgG4-RD patients. The predominant DNA methylation changes were observed in affected tissues of IgG4-RD patients with enriched “MAPK signaling pathway,” “Hippo signaling pathway,” and “VEGF signaling pathway.” Moreover, hypomethylation of IFN-related genes or enriched interferon pathways were not found in peripheral blood or salivary gland tissues of IgG4-RD patients.

The main limitation of this study is a limited sample size, especially tissue specimens, since IgG4-RD is a rare disease. Therefore, longitudinal studies are required to enlarge the sample size, and additional functional assays should be performed to fully elucidate the role of DNA methylation in IgG4-RD. Besides the current analysis of B cells, CD4^+^ T cells in IgG4-RD is a little general. It would be interesting to analyze the DNA methylation changes in some important subsets of B cells and CD4^+^ T cells, such as plasma B cells, regulatory B cells (Bregs), CD4^+^ cytotoxic T cells, Th2, follicular helper T cells (Tfh), and regulatory T cells (Tregs) in IgG4-RD patients in our future study.

## Conclusion

In conclusion, this is the first study to identify DNA methylation changes in peripheral B cells and CD4^+^ T cells, as well as salivary gland tissues from IgG4-RD patients. Our study not only demonstrated aberrant DNA methylation modifications but also emphasized the potential role of DNA methylation changes of *MBP and HLA* class, as well as other key genes and pathways in IgG4-RD pathogenesis.

## Supplementary Information

**Additional file 1: Supplementary Table 1.** Demographic and clinical characteristics of IgG4-RD patients for genome-wide DNA methylation study.

**Additional file 2: Supplementary Table 2.** The top 10 hypermethylated CpG sites in B cells of IgG4-RD patients.

**Additional file 3: Supplementary Table 3.** The top 10 hypomethylated CpG sites in B cells of IgG4-RD patients.

**Additional file 4: Supplementary Table 4.** The top 10 hypermethylated CpG sites in CD4^+ ^T cells of IgG4-RD patients.

**Additional file 5: Supplementary Table 5.** The top 10 hypomethylated CpG sites in CD4^+^ T cells of IgG4-RD patients.

**Additional file 6: Supplementary Table 6.** The top 25 hypermethylated CpG sites in salivary gland tissues of IgG4-RD patients.

**Additional file 7: Supplementary Table 7.** The top 25 hypomethylated CpG sites in salivary gland tissues of IgG4-RD patients.

**Additional file 8: Supplementary Table 8.** The DMRs in salivary gland tissues of IgG4-RD patients.

**Additional file 9: Supplementary Table 9.** The primers for pyrosequencing.

## Data Availability

Data are available upon reasonable request. Not applicable.

## Abbreviations

IgG4-RD: Immunoglobulin-G4-related disease
KEGG: Kyoto Encyclopedia of Genes and Genomes
PBMCs: Peripheral blood mononuclear cells
DMRs: Differential methylation regions
GO: Gene Ontology
DMPs: Differential methylation probes
MBP: Myelin basic protein
MS: Multiple sclerosis
TGFBR3: TGF-beta type III receptor
ITPKB: Inositol-trisphosphate 3-kinase B
RUNX1: Runt-related transcription factor 1
