# Supplementary material for: Differential CpG DNA methylation of peripheral B cells, CD4+ T cells, and salivary gland tissues in IgG4-related disease
Source: Arthritis Res Ther. 2023 Jan 7;25:4. doi: 10.1186/s13075-022-02978-5 (PMC9824958; doi:10.1186/s13075-022-02978-5)
Supplement: Supplementary file 1 — Additional file 1: Supplementary Table 1. Demographic and clinical characteristics of IgG4-RD patients for genome-wide DNA methylation study. [file 13075_2022_2978_MOESM1_ESM.docx]

**Supplementary Table 1. Demographic and clinical characteristics of IgG4-RD patients for genome-wide DNA methylation study.**

| **Variables** | **Peripheral blood** | **Tissue** |
| --- | --- | --- |
|  | **IgG4-RD**  **(n=10)** | **IgG4-RD**  **(n=4)** |
| **Age of onset (years)** | 43.50±20.21 | 56.75±6.95 |
| **Age of diagnosis (years)** | 48.10±16.82 | 57.50±6.76 |
| **Male, N (% )** | 4 (40.00%) | 0 (0.00%) |
| **Disease duration (months)** | 24.00  (5.50-120.00) | 12.00  (6.00-15.00) |
| **Number of organs involved** | 2  （1.00-3.25） | 3  (2.25-3.75) |
| **Diagnostic score*** | 27.6±8.7 | 40.50±8.39 |
| **IgG (g/L)** | 15.81±5.56 | 15.74±7.30 |
| **IgA (g/L)** | 2.04±0.82 | 2.49±1.40 |
| **IgM (g/L)** | 0.94  (0.53-1.15) | 1.07  (0.68-2.33) |
| **IgG1 (mg/L)** | 7935.00  (7355.00-9705.00) | 7745.00  (3815.00-14675.00) |
| **IgG2 (mg/L)** | 4365.00  (3430.00-6307.50) | 4865.00  (3722.50-5587.50) |
| **IgG3 (mg/L)** | 573.50  (164.00-686.00) | 526.00  (129.00-878.00) |
| **IgG4 (mg/L)** | 3140.00  (1917.50-15450.00) | 6200.00  (4320.00-12542.50) |
| **IgE (KU/L)** | 239.00  (24.35-539.50) | 74.25  (64.40-575.00) |
| **2018 RI** | 4.4±2.80 | 6.25±2.06 |
