# Supplementary material for: Differential CpG DNA methylation of peripheral B cells, CD4+ T cells, and salivary gland tissues in IgG4-related disease
Source: Arthritis Res Ther. 2023 Jan 7;25:4. doi: 10.1186/s13075-022-02978-5 (PMC9824958; doi:10.1186/s13075-022-02978-5)
Supplement: Supplementary file 2 — Additional file 2: Supplementary Table 2. The top 10 hypermethylated CpG sites in B cells of IgG4-RD patients. [file 13075_2022_2978_MOESM2_ESM.docx]

**Supplementary Table 2 The top 10 hypermethylated CpG sites in B cells of IgG4-RD patients**

| **Gene symbol** | **Gene name** | **CpG site** | **CHR** | **CpG island** | **Gene property** | **deltaBeta** | **P.Value** |
| --- | --- | --- | --- | --- | --- | --- | --- |
| IQCK | IQ domain-containing protein K | cg03615426 | 16 | opensea | Body | 0.22 | 0.011 |
| IQCK | IQ domain-containing protein K | cg10266221 | 16 | opensea | Body | 0.22 | 0.010 |
| UMODL1 | Uromodulin-like 1 precursor | cg09727148 | 21 | opensea | 3’UTR | 0.21 | 0.041 |
| AHDC1 | AT-Hook DNA binding motif containing 1 | cg00178877 | 1 | shore | Body | 0.21 | 0.017 |
| LARS2 | Leucyl-TRNA Synthetase 2 | cg14416782 | 3 | opensea | Body | 0.21 | 0.045 |
| RASA3 | RAS P21 Protein Activator 3 | cg03646740 | 13 | shore | Body | 0.20 | 0.003 |
| USP16 | Ubiquitin Specific Peptidase 16 | cg23403192 | 21 | shore | TSS1500 | 0.19 | 0.035 |
| BTBD11 | BTB Domain Containing 11 | cg01245561 | 12 | opensea | Body | 0.16 | 0.014 |
| CLECL1 | C-type lectin-like domain family 1 | cg15772839 | 12 | opensea | 3’UTR | 0.16 | 0.0004 |
| LOC441897 | LOC441897 | cg18346412 | 1 | opensea | Body | 0.16 | 0.002 |
| ABCC13 | ABC transporter C family member 13 | cg04985582 | 21 | opensea | TSS200 | 0.16 | 0.023 |

CHR: Chromosome.
