# Supplementary material for: Differential CpG DNA methylation of peripheral B cells, CD4+ T cells, and salivary gland tissues in IgG4-related disease
Source: Arthritis Res Ther. 2023 Jan 7;25:4. doi: 10.1186/s13075-022-02978-5 (PMC9824958; doi:10.1186/s13075-022-02978-5)
Supplement: Supplementary file 3 — Additional file 3: Supplementary Table 3. The top 10 hypomethylated CpG sites in B cells of IgG4-RD patients. [file 13075_2022_2978_MOESM3_ESM.docx]

**Supplementary Table 3 The top 10 hypomethylated CpG sites in B cells of IgG4-RD patients**

| **Gene symbol** | **Gene name** | **CpG site** | **CHR** | **CpG island** | **Gene property** | **deltaBeta** | **P.Value** |
| --- | --- | --- | --- | --- | --- | --- | --- |
| TMEM9B | Transmembrane protein 9B | cg15570860 | 11 | shore | TSS1500 | -0.31 | 0.024 |
| CLIC6 | Chloride Intracellular Channel 6 | cg12080266 | 21 | opensea | Body | -0.28 | 0.026 |
| MIR492 | MicroRNA 492 | cg08922308 | 12 | opensea | TSS1500 | -0.21 | 0.010 |
| ARNTL | Aryl hydrocarbon receptor neclear translocator like | cg13286116 | 11 | shore | 5'UTR | -0.18 | 0.009 |
| TNN | Tenascin N | cg23658987 | 1 | shore | Body | -0.17 | 0.022 |
| MBP | Myelin basic protein | cg18455083 | 18 | shelf | Body | -0.16 | 0.036 |
| CDK15 | Cyclin-dependent kinase 15 | cg17851604 | 2 | opensea | Body | -0.16 | 0.002 |
| PIGL | Phosphatidylinositol glycan anchor biosynthesis class L | cg06338664 | 17 | opensea | Body | -0.15 | 0.002 |
| HLA-DRB1 | Major histocompatibility complex, Class II, DR Beta 1 | cg08269402 | 6 | shelf | Body | -0.15 | 0.035 |
| MYO1D | Myosin ID | cg18200150 | 17 | shore | Body | -0.15 | 0.002 |

CHR: Chromosome.
