# Supplementary material for: Differential CpG DNA methylation of peripheral B cells, CD4+ T cells, and salivary gland tissues in IgG4-related disease
Source: Arthritis Res Ther. 2023 Jan 7;25:4. doi: 10.1186/s13075-022-02978-5 (PMC9824958; doi:10.1186/s13075-022-02978-5)
Supplement: Supplementary file 4 — Additional file 4: Supplementary Table 4. The top 10 hypermethylated CpG sites in CD4+ T cells of IgG4-RD patients. [file 13075_2022_2978_MOESM4_ESM.docx]

**Supplementary Table 4 The top 10 hypermethylated CpG sites in CD4^+^ T cells of IgG4-RD patients**

| **Gene symbol** | **Gene name** | **CpG site** | **CHR** | **CpG island** | **Gene property** | **deltaBeta** | **P.Value** |
| --- | --- | --- | --- | --- | --- | --- | --- |
| MAST4 | Microtubule Associated Serine/Threonine Kinase Family Member 4 | cg12145624 | 5 | shore | TSS1500 | 0.39 | 0.013 |
| AHDC1 | AT-Hook DNA Binding Motif Containing 1 | cg00178877 | 1 | shore | Body | 0.21 | 0.021 |
| BICC1 | BicC Family RNA Binding Protein 1 | cg12342675 | 10 | opensea | Body | 0.21 | 0.001 |
| USP16 | Ubiquitin Specific Peptidase 16 | cg23403192 | 21 | shore | TSS1500 | 0.18 | 0.049 |
| IQCK | IQ domain-containing protein K | cg03615426 | 16 | opensea | Body | 0.17 | 0.020 |
| LOC727677 | LOC727677 | cg08682625 | 8 | opensea | Body | 0.17 | 0.006 |
| MYO1D | Myosin 1D | cg24550644 | 17 | island | Body | 0.16 | 0.003 |
| LOC441897 | LOC441897 | cg18346412 | 1 | opensea | Body | 0.15 | 0.004 |
| CAV2 | Caveolin 2 | cg00917413 | 7 | shore | TSS1500 | 0.14 | 0.028 |
| IQCK | IQ domain-containing protein K | cg10266221 | 16 | opensea | Body | 0.14 | 0.008 |
| TGFBR2 | Transforming Growth Factor Beta Receptor 2 | cg16918625 | 3 | opensea | Body | 0.14 | 0.001 |

CHR: Chromosome.
