# Supplementary material for: Differential CpG DNA methylation of peripheral B cells, CD4+ T cells, and salivary gland tissues in IgG4-related disease
Source: Arthritis Res Ther. 2023 Jan 7;25:4. doi: 10.1186/s13075-022-02978-5 (PMC9824958; doi:10.1186/s13075-022-02978-5)
Supplement: Supplementary file 5 — Additional file 5: Supplementary Table 5. The top 10 hypomethylated CpG sites in CD4+ T cells of IgG4-RD patients. [file 13075_2022_2978_MOESM5_ESM.docx]

**Supplementary Table 5 The top 10 hypomethylated CpG sites in CD4^+^ T cells of IgG4-RD patients**

| **Gene symbol** | **Gene name** | **CpG site** | **CHR** | **CpG island** | **Gene property** | **deltaBeta** | **P.Value** |
| --- | --- | --- | --- | --- | --- | --- | --- |
| TMEM9B | Transmembrane protein 9B | cg15570860 | 11 | shore | TSS1500 | -0.30 | 0.021 |
| CLIC6 | Chloride Intracellular Channel 6 | cg12080266 | 21 | opensea | Body | -0.28 | 0.022 |
| CCS | Copper Chaperone For Superoxide Dismutase | cg24851651 | 11 | shelf | Body | -0.27 | 0.032 |
| PAWR | Pro-Apoptotic WT1 Regulator | cg11258982 | 12 | opensea | Body | -0.24 | 0.026 |
| FIP1L1 | Factor Interacting With PAPOLA And CPSF1 | cg17483361 | 4 | island | 5’UTR | -0.24 | 0.001 |
| ARHGAP15 | Rho GTPase Activating Protein 15 | cg00640622 | 2 | opensea | Body | -0.20 | 0.000 |
| ARHGAP26 | Rho GTPase Activating Protein 26 | cg21591141 | 5 | opensea | Body | -0.20 | 0.003 |
| ARHGAP26 | Rho GTPase Activating Protein 26 | cg12655260 | 5 | opensea | Body | -0.19 | 0.001 |
| PKD1L1 | Polycystin 1 Like 1, Transient Receptor Potential Channel Interacting | cg13840476 | 7 | opensea | Body | -0.17 | 0.007 |
| HLA-DRB1 | Major histocompatibility complex, Class II, DR Beta 1 | cg08269402 | 6 | shelf | Body | -0.17 | 0.021 |
| HLA-DQB2 | Major histocompatibility complex, Class II, DQ Beta 2 | cg07180897 | 6 | shore | Body | -0.16 | 0.019 |

CHR: Chromosome.
