# Supplementary material for: Differential CpG DNA methylation of peripheral B cells, CD4+ T cells, and salivary gland tissues in IgG4-related disease
Source: Arthritis Res Ther. 2023 Jan 7;25:4. doi: 10.1186/s13075-022-02978-5 (PMC9824958; doi:10.1186/s13075-022-02978-5)
Supplement: Supplementary file 6 — Additional file 6: Supplementary Table 6. The top 25 hypermethylated CpG sites in salivary gland tissues of IgG4-RD patients. [file 13075_2022_2978_MOESM6_ESM.docx]

**Supplementary Table 6 The top 25 hypermethylated CpG sites in salivary gland tissues of IgG4-RD patients**

| **Gene symbol** | **Gene name** | **CpG site** | **CHR** | **CpG island** | **Gene property** | **deltaBeta** | **P.Value** |
| --- | --- | --- | --- | --- | --- | --- | --- |
| LOH12CR1 | BLOC-1-related complex subunit 5 | cg18113757 | 12 | opensea | Body | 0.73 | 0.0003 |
| PFKP | Phosphofructokinase, Platelet | cg12802403 | 10 | shore | Body | 0.72 | 0.0003 |
| ARSB | Arylsulfatase B | cg05516344 | 5 | opensea | Body | 0.71 | 0.0004 |
| SNX29 | Sorting Nexin 29 | cg16037106 | 16 | opensea | Body | 0.71 | 0.0003 |
| TBCD | Tubulin Folding Cofactor D | cg07409471 | 17 | shelf | Body | 0.71 | 0.0004 |
| PFKP | Phosphofructokinase, Platelet | cg03125474 | 10 | shore | Body | 0.71 | 0.0003 |
| STARD13 | StAR Related Lipid Transfer Domain Containing 13 | cg14491582 | 13 | opensea | 5’UTR | 0.71 | 0.0003 |
| GALNT10 | Polypeptide N-Acetylgalactosaminyltransferase 10 | cg09607085 | 5 | opensea | Body | 0.71 | 0.0003 |
| EIF4E3 | Eukaryotic Translation Initiation Factor 4E Family Member 3 | cg09606770 | 3 | opensea | Body | 0.71 | 0.0003 |
| VGLL4 | Vestigial Like Family Member 4 | cg00691123 | 3 | opensea | Body | 0.71 | 0.0003 |
| TGFBR3 | Transforming Growth Factor Beta Receptor 3 | cg17468459 | 1 | opensea | Body | 0.71 | 0.0004 |
| GLG1 | Golgi Glycoprotein 1 | cg24381100 | 16 | opensea | ExonBnd | 0.71 | 0.0003 |
| RBM34 | RNA Binding Motif Protein 34 | cg06399398 | 1 | opensea | Body | 0.70 | 0.0004 |
| EPAS1 | Endothelial pAS domain protein 1 | cg20922371 | 2 | opensea | Body | 0.69 | 0.0004 |
| ITPKB | Inositol-trisphosphate 3-Kinase B | cg13962718 | 1 | shore | Body | 0.69 | 0.0004 |
| TBCD | Tubulin Folding Cofactor D | cg10862567 | 17 | opensea | Body | 0.69 | 0.0003 |
| TSC22D1 | TSC22 Domain Family Member 1 | cg17600231 | 13 | shelf | 3’UTR | 0.69 | 0.0003 |
| SLC44A2 | Solute Carrier Family 44 Member 2 | cg21631439 | 19 | shore | Body | 0.69 | 0.0003 |
| TRIOBP | TRIO And F-Actin Binding Protein | cg00086800 | 22 | opensea | Body | 0.69 | 0.0003 |
| ACAD11 | Acyl-CoA Dehydrogenase Family Member 11 | cg00981413 | 3 | opensea | Body | 0.69 | 0.0005 |
| KCNAB2 | Potassium Voltage-Gated Channel Subfamily A Regulatory Beta Subunit 2 | cg01184901 | 1 | shore | Body | 0.69 | 0.0004 |
| PDE4D | Phosphodiesterase 4D | cg13152606 | 5 | opensea | 1stExon | 0.69 | 0.0004 |
| RERE | Arginine-Glutamic Acid Dipeptide Repeats | cg14607332 | 1 | opensea | Body | 0.69 | 0.0004 |
| ARID1B | AT-Rich Interaction Domain 1B | cg15758896 | 6 | opensea | Body | 0.69 | 0.0004 |
| UAP1 | UDP-N-Acetylglucosamine Pyrophosphorylase 1 | cg13178361 | 1 | shore | 5’UTR | 0.68 | 0.0003 |
| LGALS3 | Galectin 3 | cg06001396 | 14 | opensea | 5’UTR | 0.68 | 0.0003 |
| LOC102724933 | LOC102724933 | cg00201393 | 12 | opensea | TSS200 | 0.68 | 0.0004 |

CHR: Chromosome.
