# Supplementary material for: Differential CpG DNA methylation of peripheral B cells, CD4+ T cells, and salivary gland tissues in IgG4-related disease
Source: Arthritis Res Ther. 2023 Jan 7;25:4. doi: 10.1186/s13075-022-02978-5 (PMC9824958; doi:10.1186/s13075-022-02978-5)
Supplement: Supplementary file 7 — Additional file 7: Supplementary Table 7. The top 25 hypomethylated CpG sites in salivary gland tissues of IgG4-RD patients. [file 13075_2022_2978_MOESM7_ESM.docx]

**Supplementary Table 7 The top 25 hypomethylated CpG sites in salivary gland tissues of IgG4-RD patients**

| **Gene symbol** | **Gene name** | **CpG site** | **CHR** | **CpG island** | **Gene property** | **deltaBeta** | **P.Value** |
| --- | --- | --- | --- | --- | --- | --- | --- |
| CD69 | CD69 | cg07354583 | 12 | opensea | Body | -0.73 | 0.0003 |
| RUNX1 | Runt-related transcription factor-1 | cg04915566 | 21 | opensea | 5’UTR | -0.73 | 0.0003 |
| TMEM229B | Transmembrane protein 229B | cg11657366 | 14 | opensea | 5’UTR | -0.72 | 0.0003 |
| JAZF1 | Juxtaposed with another zinc finger protein 1 | cg11562379 | 7 | opensea | Body | -0.72 | 0.0003 |
| SLC7A6 | Solute Carrier Family 7 Member 6 | cg07688618 | 16 | opensea | Body | -0.72 | 0.0003 |
| FAM69A | Family with sequence similarity 69, member A | cg12632472 | 1 | opensea | Body | -0.72 | 0.0003 |
| EVL | Enah/Vasp-like | cg20256286 | 14 | opensea | Body | -0.72 | 0.0003 |
| CYTIP | Cytohesin 1 Interacting Protein | cg14678223 | 2 | opensea | TSS1500 | -0.72 | 0.0003 |
| WIPF1 | WAS/WASL Interacting Protein Family Member 1 | cg11526752 | 2 | opensea | 5’UTR | -0.72 | 0.0003 |
| CYTIP | Cytohesin 1 Interacting protein | cg24848491 | 2 | opensea | TSS1500 | -0.72 | 0.0003 |
| CYTIP | Cytohesin 1 Interacting protein | cg10559416 | 2 | opensea | 1stExon | -0.71 | 0.0003 |
| TNFSF8 | TNF Superfamily Member 8 | cg18390596 | 9 | opensea | Body | -0.71 | 0.0003 |
| LOC102724 | LOC102724 | cg25817655 | 1 | shelf | Body | -0.71 | 0.0003 |
| NCKAP1L | NCK Associated Protein 1 Like | cg21376733 | 12 | opensea | 1stExon | -0.71 | 0.0003 |
| SETBP1 | SET Binding Protein 1 | cg07856430 | 18 | opensea | Body | -0.70 | 0.0003 |
| KIAA0748 | Thymocyte expressed, positive selection associated 1 | cg19219778 | 12 | opensea | TSS200 | -0.70 | 0.0003 |
| PPP1R16B | Protein Phosphatase 1 Regulatory Subunit 16B | cg27377213 | 20 | shore | TSS1500 | -0.70 | 0.0003 |
| GRAP2 | GRB2 Related Adaptor Protein 2 | cg03840259 | 22 | opensea | TSS200 | -0.70 | 0.0003 |
| CASP8 | Caspase 8 | cg26842802 | 2 | opensea | 5’UTR | -0.70 | 0.0003 |
| ARSG | Arylsulfatase G | cg14921479 | 17 | opensea | Body | -0.70 | 0.0003 |
| ITGB2-AS1 | Integrin Subunit Beta 2-Antisense RNA 1 | cg16703541 | 21 | opensea | TSS1500 | -0.70 | 0.0003 |
| MGAT1 | Alpha-1,3-Mannosyl-Glycoprotein 2-Beta-N-Acetylglucosaminyltransferase | cg20399011 | 5 | shore | TSS1500 | -0.70 | 0.0003 |
| CD37 | CD37 | cg15046675 | 19 | shelf | 1stExon | -0.69 | 0.0003 |
| MAP2K2 | Mitogen-Activated Protein Kinase Kinase 2 | cg12092651 | 19 | opensea | Body | -0.69 | 0.0003 |
| WIPF1 | WAS/WASL Interacting Protein Family Member 1 | cg10725892 | 2 | opensea | 5’UTR | -0.69 | 0.0003 |
| TNFAIP8L2 | Tumor Necrosis Factor, Alpha-induced Protein Like 2 | cg23612220 | 1 | opensea | 5’UTR | -0.69 | 0.0003 |
| RCSD1 | RCSD Domain Containing 1 | cg25765104 | 1 | shore | Body | -0.69 | 0.0003 |
| EVL | Enah/Vasp-like | cg19918549 | 14 | opensea | 1stExon | -0.69 | 0.0003 |

CHR: Chromosome.
