# Supplementary material for: Differential CpG DNA methylation of peripheral B cells, CD4+ T cells, and salivary gland tissues in IgG4-related disease
Source: Arthritis Res Ther. 2023 Jan 7;25:4. doi: 10.1186/s13075-022-02978-5 (PMC9824958; doi:10.1186/s13075-022-02978-5)
Supplement: Supplementary file 8 — Additional file 8: Supplementary Table 8. The DMRs in salivary gland tissues of IgG4-RD patients. [file 13075_2022_2978_MOESM8_ESM.docx]

**Supplementary Table 8 The DMRs in salivary gland tissues of IgG4-RD patients**

|  | **seqnames** | **no.cpgs** | **maxbetafc** | **meanbetafc** | **overlapping.promoters** |
| --- | --- | --- | --- | --- | --- |
| DMR_1 | chr7 | 59 | 0.66 | 0.40 | HOXA3-002, HOXA4-001, HOXA3-001, HOXA4-201,  HOXA4-004, HOXA-AS2-001, HOXA-AS2-005,  HOXA-AS3-003, HOXA-AS2-003, HOXA-AS2-002,  HOXA-AS2-004, HOXA-AS2-007 |
| DMR_2 | chr7 | 69 | 0.64 | 0.39 | HOXA5-001, HOXA6-001, HOXA-AS3-001, HOXA5-002,  HOXA-AS3-004, HOXA-AS3-005, HOXA3-006, HOXA3-007,  HOXA3-005, HOXA-AS3-002 |
| DMR_3 | chr11 | 34 | -0.60 | -0.39 | TSPAN32-001, TSPA32-004, TSPAN32-014, TSPAN32-005,  C11orf21-001, TSPAN32-007, TSPAN32-002, TSPAN32-003,  C11orf21-004, TSPAN32-006, C11orf21-002, TSPAN32-012,  TSPAN32-019, TSPAN32-011, TSPAN32-008, TSPAN32-010,  C11orf21-005, TSPAN32-009 |
| DMR_4 | chr7 | 28 | 0.64 | 0.48 | HOXA-AS2-006, HOXA-AS2-011, HOXA-AS2-008,  HOXA3-004, HOXA-AS2-012 |
| DMR_5 | chr15 | 36 | -0.61 | -0.35 | NA |
| DMR_6 | chr17 | 29 | 0.67 | 0.41 | HOXB2-001, HOXB-AS2-001, HOXB-AS3-012, HOXB3-001,  HOXB3-012, HOXB3-011, HOXB-AS1-004 |
| DMR_7 | chr17 | 21 | -0.69 | -0.46 | BZRAP1-001, BZRAP1-002, BZRAP1-201, MIR142-201,  BZRAP1-AS1-003, MIR142-001, BZRAP1-AS1-002,  BZRAP1-AS1-009 |
| DMR_8 | chr17 | 47 | 0.59 | 0.29 | HOXB6-002, HOXB7-001, HOXB7-003, HOXB6-003,  HOXB6-001, HOXB6-004, HOXB6-AS3-003, HOXB6-AS3-001,  HOXB3-013 |
| DMR_9 | chr16 | 25 | -0.62 | -0.34 | LAT-201, LAT-001, LAT-004, LAT-002, LAT-007, LAT-008,  LAT-006, LAT-009, LAT-012, LAT-015, LAT-005, LAT-003,  LAT-013, LAT-010, LAT-014, LAT-011 |
| DMR_10 | chr1 | 25 | 0.46 | 0.32 | S100A1-005, S100A1-003, S100A13-202, S100A13-203,  S100A1-004, S100A13-204, S100A1-002, S100A13-002,  S100A13-006, RP1-178F15.4-202 |

CHR: Chromosome.
