# Supplementary material for: Differential CpG DNA methylation of peripheral B cells, CD4+ T cells, and salivary gland tissues in IgG4-related disease
Source: Arthritis Res Ther. 2023 Jan 7;25:4. doi: 10.1186/s13075-022-02978-5 (PMC9824958; doi:10.1186/s13075-022-02978-5)
Supplement: Supplementary file 9 — Additional file 9: Supplementary Table 9. The primers for pyrosequencing. [file 13075_2022_2978_MOESM9_ESM.docx]

**Supplementary Table 9. The primers for pyrosequencing.**

| **CpG** | Primers | Sequence (5' to 3') | **5' end modification** |
| --- | --- | --- | --- |
| CG18455083 | Forward PCR primer | TTGTGAGGAGATGGGAGAGTT |  |
|  | Reverse PCR primer | CCTCTATTTATATCTTAATTTCCCCATAC | 5'-Biotin |
|  | Sequencing primer | GGAGATGGGAGAGTTT |  |
| CG08269402 | Forward PCR primer | TGTAGGGGTTGGGTTTTTGAA |  |
|  | Reverse PCR primer | CTATCTTCCTAACTCATTCCCTCTA | 5'-Biotin |
|  | Sequencing primer | GGTTGGATTAGGAGAAAA |  |
| CG07180897 | Forward PCR primer | GGGTGGGGTAGTGTTAGT |  |
|  | Reverse PCR primer | CCATCCACCTTATCCTCTACTCTACAT | 5'-Biotin |
|  | Sequencing primer | GTATTAGGGTAGAAAGAATTGTTTA |  |
